# Whole-genome selective sweep analysis of Danish Large White and Chinese indigenous pig populations

**DOI:** 10.1080/10495398.2025.2467411

**Published:** 2025-03-01

**Authors:** Xudong Wu, Decai Xiang, Bofang Duan, Wei Zhang, Mo. Li, Yueyun Ding, Zilong Zhao, Guiying Zhao, Zongjun Yin

**Affiliations:** ^a^ Anhui Provincial Key Laboratory of Livestock and Poultry Product Safety Engineering, Institute of Animal Husbandry and Veterinary Medicine, Anhui Academy of Agricultural Sciences, P.R. China; ^b^ Yunnan Provincial Engineering Laboratory of Animal Genetic Resource Conservation and Germplasm Enhancement, Yunnan Animal Science and Veterinary Institute, P.R. China; ^c^ Yunnan Animal Disease Prevention and Control Center, P.R. China; ^d^ College of Animal Science and Technology, Anhui Agricultural University, P.R. China; ^e^ Anhui Da Zi Ran Pig Breeding Farm Corporation Limited, P.R. China; ^f^ College of Animal Science and Technology, Yunnan Agricultural University, P.R. China

**Keywords:** Signatures of selection, pig breeding, whole-genome re-sequencing, reproduction trait, candidate gene

## Abstract

The pig industry is an important component of Chinese agriculture. Chinese indigenous and Western commercial pig breeds provide valuable genetic resources for sustainable development of the pig industry, and selective signal analysis can advance our understanding of artificial and natural selective processes. In this study, we used whole genome re-sequencing data to analyze selection signatures in 43 Danish Large White [LW] pigs and 60 Chinese indigenous pigs (24 Anqing six-end white [AQ], 6 Asian wild [SS] pigs, 15 Diqing Tibetan [DQZ], and 15 Diannan small-ear [DN] pigs). We employed two calculation methods (F_ST_ and π ratio) to identify the selection signals of LW and Chinese indigenous pigs (top 1%). Among the selective sweep regions, 15 and 117 candidate genes were identified in Chinese indigenous pigs and LW pigs, respectively. The *PIK3IP1*, *TUG1*, and *SELENOM* genes were related to environmental adaptation in Chinese indigenous pigs; *ALDH1A2, APC2*, *PTBP1*, *APQ9*, and *FGF7* were related to reproductive performance in LW pigs. Our findings provide insights into the genetic basis of economic traits of LW and Chinese indigenous pigs and offer a useful reference for future pig breeding and production.

## Introduction

Genomic research in pigs has been pivotal in revolutionizing various aspects of swine production, breeding, and evolutionary studies. Advancements in sequencing technologies and bioinformatics tools have facilitated a deeper understanding of the complexity of the pig genome and its implications for agriculture and biomedicine.^1^^,^^2^ Identifying genetic variations and genotypic associations with key economic traits are essential for pig breeding and production, and high-quality reference genomes are essential in genomics studies.^3^ The pig reference genome sequence (Sscrofa11.1) aids in the accurate identification of genetic variation.^4–5^

Genome-wide selective sweep analysis allows researchers to delve into the intricate evolutionary dynamics that shape populations.^6^ This method provides valuable insights into how artificial or natural selection influences genetic variability within a special genome. One of the key strengths of this analytical approach is its ability to identify genomic regions that have undergone rapid and recent selective pressures.^7^ By pinpointing these regions, researchers can unravel the genetic basis of adaptation and evolution, shedding light on the mechanisms driving species divergence and adaptation to diverse environments. Moreover, genome-wide selective sweep analysis facilitates the identification of candidate genes associated with specific phenotypic traits or adaptations, and can reveal the functional significance of these genetic changes. Selective regions comprising genes related to olfactory receptors and oxidoreductases were identified in Landrace and York-shire pigs, suggesting that olfactory function is a key trait involved in the domestication of these breeds.^8^ According to Gao et al., that nucleic acid polymorphisms (θπ) of the *MSRB3* gene exhibited a significant decrease in the Shanghai local pig breed population when compared with the commercial pig (Duroc, Landrace, and Yorkshire) population. However, the *MSRB3* gene exerts a significant selective effect in the Shanghai local pig population, which is related to the long lop ear phenotype.^9^

There are significant phenotypic differences in meat quality, adaptability, and production performance between LW and Chinese local pig breeds, owing to centuries of evolution and breeding.^10^^,^^11^ In a previous study, we detected genome-wide genomic variation differences between Danish LW and Chinese indigenous pigs, and a total of 1,289 breed-specific non-synonymous SNPs were obtained in LW pigs.^12^ However, there remains a need to understand how domestication and artificial selection have shaped the pig genome to facilitate the promotion of economic traits in pigs and support the sustainable development of the pig industry. In this study, we used genetic differentiation (F_ST_) and pairwise nucleotide variation (θπ ratio) as the measure of variability to detect the selective sweep of Danish LW and Chinese indigenous pigs (Anqing six white pigs [AQ], Asian sus scrofa [SS], Diqing Tibetan pig [DQT], and Diannan small ear pigs [DN]). Our results provide a new insights into the genetic differences between these two populations; moreover, the selective regions and candidate genes identified herein offer potential markers for future pig breeding and production.

## Materials and methods

The Genetic variation information of 103 pigs was determined, including 43 Danish LW pigs and 60 Chinese indigenous pigs (24 Anqing six-end white [AQ], 6 Asian wild [SS] pigs, 15 Diqing Tibetan [DQZ], and 15 Diannan small-ear [DN] pigs). The data collection and sequencing methods for all samples can be obtained from our previous studies.^12^^,^^13^ This study did not involve animal experiments and therefore did not require approval from an Animal Ethics Committee. The comparison software bwa (http://bio-bwa.sourceforge.net/) was used to compare the data to the reference genome (Sscrofa11.1), and the comparison SAMtools software (https://github.com/samtools/samtools) was used to sort the results and label the repeat sequences.^14^^,^^15^ We used the GATK software (https://gatk.broadinstitute.org) to identify SNP and Indel sites, and the ANNOVAR software (https://annovar.openbioinformatics.org/) to annotate the detected SNPs, Indels, and other genomic variations with external databases.^16^^,^^17^

All China pig populations were combined into one group (non-LW), and compared with the LW pigs. The original SNPs were filtered to improve the accuracy of the analysis; the filtering conditions were as follows: (i) missing rate of marker sites > 20%, (ii) minor allele frequency (MAF) ≥ 0.1%, and (iii) removal of marker sites in sex chromosomes. Based on the filtered SNPs, the PopGenome software was used to slide the window according to the physical length, with 100 kb as the window and 10 kb as the step length, and F_ST_, and θπ ratio tests were conducted.^18^ Overlapping window regions between F_ST_ and θπ ratio analysis with 1% level were considered as the selection signature, from which candidate genes in sweep regions were detected based on the scan results. An online tool (https://www.omicshare.com /tools/) was used to perform Gene Ontology (GO) and Kyoto Encyclopedia of Genes and Genomes (KEGG) analyses of identified genes. Subsequently, the published pig QTL information was downloaded from the pig quantitative trait loci (QTL) database (https://www.animalgenome.org/cgi-bin/ QTLdb/SS/index, Sscrofa11.1), the QTL database used in this study was last updated on 28 April 2024, and overlapped with identified selection regions of LW and Chinese indigenous pigs. All QTLs were classified into five groups according to the associated traits: exterior, production, meat and carcass, reproduction, and health. The QTL filtration parameters and overlapping methods are based on the descriptions of Zhang et al.^19^ Finally, an extensive literature review was conducted to gather information for exploratory investigations on gene function.

## Results

### Genome-wide scanning for selection signatures

**Figure 1.**
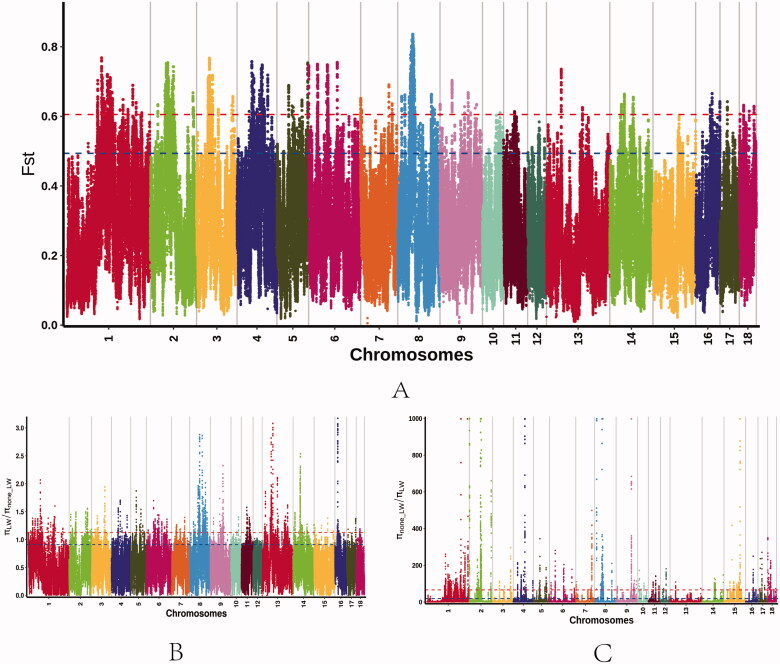
Identification of genomic regions with selection sweep in the LW and non-LW pig populations, calculated using a 100-kb sliding window approach with 10 kb step-size. (**a**) Distribution of F_ST_ values among autosomal chromosomes. The grey line represents the 0.01 level. (**b**) Distribution of the θπ ratio among autosomal chromosomes (non-LW/LW). The grey line represents the 0.01 level. (**c**) Distribution of the θπ ratio among autosomal chromosomes (LW/non-LW). The grey line represents the 0.01 level.

**Figure 2.**
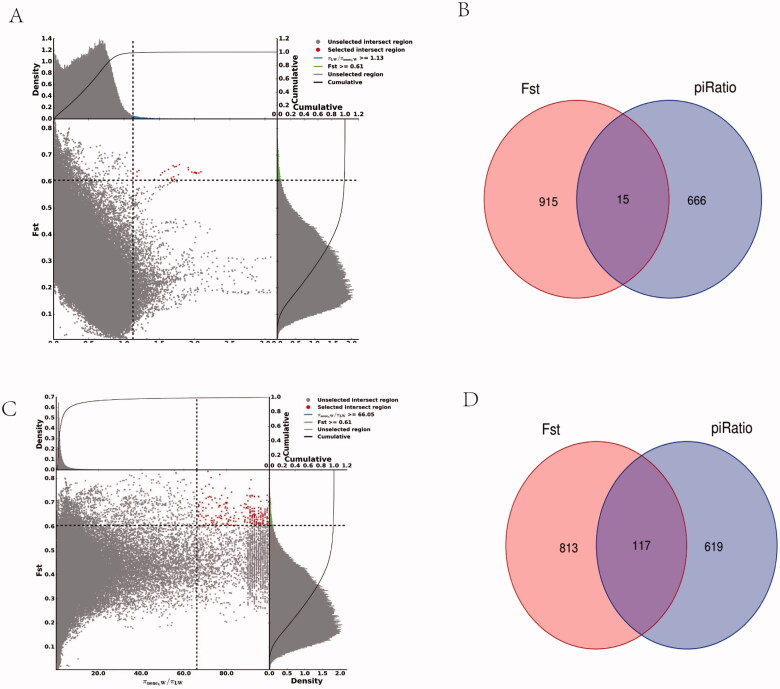
Final selection regions in non-LW and LW pig populations based on two statistics. (**a**) Final selection regions in non-LW pigs based on two statistics (Points located to the right of the vertical dashed lines and above the horizontal dashed line). (**b**) Number of identified genes for the final selection regions of non-LW pigs. (**c**) Final selection regions in LW pigs based on two statistics (points located to the right of the vertical dashed lines and above the horizontal dashed line). (**d**) number of identified genes of the final selection regions of LW pigs.

After variant calling and subsequent stringent quality filtering, a total of 334,696,381 high-quality SNPs were retained (12,293,061 detected only in LW; 22,403,320 detected only in non-LW; 11,486,887 detected both in LW and non-LW). From F_ST_ analysis, 2,264 selective sweep regions were identified in the genome (top 1% level, F_ST_ value ≥ 0.61; Fig. 1). From θπ ratio analysis, 2,264 selective sweep regions were identified in non-LW pigs (top 1% level, θπ value ≥ 1.13), and 2,290 selective sweep regions were identified in LW pigs (top 1% level, θπ value ≥ 66.05) (Fig. 1). Subsequently, the F_ST_ and θπ ratio analyses were combined for genome-wide selective sweep detection with the top 1% level. For the non-LW group, 28 selective sweep regions were identified with extremely high F_ST_ values and significantly high θπ ratios (Fig. 2, Table S1). These regions were distributed on Sus scrofa chromosome (SSC) 1, 5, and 14, with 25 regions observed on SSC14. Furthermore, these selective sweep regions harbored 15 candidate genes. For the LW group, 257 selective sweep regions were identified, which were distributed on SSC 1, 2, 4, 5, 7, 8, and 16, with 128 regions observed on SSC8. These selective sweep regions harbored 117 candidate genes (Fig. 2, Table S2).

### Enrichment and QTL overlap analysis of selected regions

**Figure 3.**
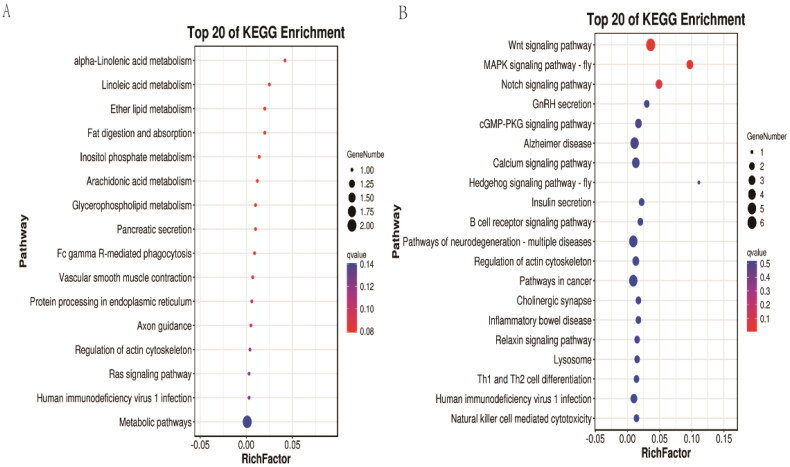
Functional analysis of candidate genes in selective sweep regions. (**a**) KEGG analysis of candidate genes of non-LW pigs. (**b**) KEGG analysis of candidate genes of LW pigs.

Candidate genes in the selective sweep regions were subjected to functional enrichment analysis. In non-LW genome selective regions, 15 candidate genes were enriched in 779 terms, which were divided into three categories: molecular function (MF; 87 terms), cellular component (CC; 81 terms), and biological process (BP; 611 terms). The significantly enriched terms were as follows: GO:0034485 phosphatidylinositol-3,4,5-trisphosphate 5-phosphatase activity, GO:0036313 phosphatidylinositol 3-kinase catalytic subunit binding, GO:0052659 inositol-1,3,4,5-tetrakisphosphate 5-phosphatase activity, GO:0052743 inositol tetrakisphosphate phosphatase activity, GO:0004445 inositol polyphosphate 5-phosphatase activity, GO:0034594 phosphatidylinositol trisphosphate phosphatase activity, GO:0034594 phosphatidylinositol trisphosphate phosphatase activity, and GO:0046030 inositol trisphosphate phosphatase activity (q value < 0.05) (Table S3). In LW genome selective regions, 117 candidate genes were enriched in 2049 GO terms, which were divided into three categories: molecular function (MF; 316 terms), cellular component (CC; 227 terms) and biological process (BP; 1506 terms). The significantly enriched terms were as follows: GO:0007212 dopamine receptor signaling pathway, GO:1903350 response to dopamine, GO:1903351 cellular response to dopamine, GO:0071867 response to monoamine, GO:0071868 cellular response to monoamine stimulus, GO:0071869 response to catecholamine, and GO:0071870 cellular response to catecholamine stimulu (q value < 0.05) (Table S4). Subsequently, KEGG enrichment analysis was performed on the candidate genes from the selective sweep regions. For non-LW genome selective regions, 16 pathways displayed enrichment, among which the KO:00592: alpha-linolenic acid metabolism path-way, KO:00591 linoleic acid metabolism pathway, KO:00565 ether lipid metabolism pathway, and KO:04975 fat digestion and absorption pathway were enriched (Fig. 3). For LW genome selective regions, 113 pathways were enriched, among which the KO:04310 WNT signaling pathway and KO:04013 MAPK signaling pathway were significantly enriched (q value < 0.05) (Fig. 3). We identified 11 QTL overlapping regions in the non-LW group, comprising 29 QTL loci; most QTLs were localized in health traits, accounting for 41.38% of all loci, and were concentrated in blood parameters, disease susceptibility, and immune capacity (Table S5). For LW genome selective sweep regions, 87 QTL overlapping regions were identified, containing 116 QTL loci; among them, 10 QTL loci were associated with reproduction traits and were concentrated in the corpus luteum number, gestation length, number of mummified pigs, and teat number (Table S6). Furthermore, after an extensive literature review, several genes were found to be associated with the characteristics of LW and non-LW pigs. Three genes were related to environmental adaptation in non-LW pigs (*PIK3IP1*, *TUG1*, *SELENOM*), and the *ALDH1A2*, *APC2*, *PTBP1*, *APQ9*, and *FGF7* genes were related to reproductive performance in LW pigs.

## Discussion

The long-term processes of artificial and natural selection have resulted in the creation of several genetic footprints within the genomes of diverse pig breeds. Various comparative studies examine the genetic diversity and selection signatures in various pig breeds, including Chinese indigenous pig and commercial pig breeds. In conjunction with F_ST_ and the θ_π_ ratio methods for analyzing selection signal between six Chinese indigenous pig breeds and four commercial pig breeds, Zhang et al. identified specific genomic regions associated with domestication characteristics of indigenous pigs.^20^ In another study on genomic selection signals in indigenous and commercial pigs, it was found that the *DKK2* gene, which is associated with meat quality, was subjected to disparate selection pressures.^21^ These studies and our findings will further enhance our understanding of the genetic differences among different breeds.

### Breeding objectives for chinese indigenous pigs from population-specific selective signatures

Among the non-LW group, there were three prominent Chinese indigenous pig breeds. DQZ is a typical plateau-type breed from Yunnan Province that has evolved unique biological characteristics with long-term natural selection for adapting to low temperatures, low-oxygen levels, low pressure, and strong ultraviolet radiation; furthermore, it has high disease resistance.^22^ DN inhabits the south of Yunnan Province and has exceptional resistance to coarse foods, high temperatures, and humidity.^23^ AQ is a famous native pig breed in Yangtze River basin, and exhibits excellent adaptive capacity and good meat quality. Our previous study found that genome homozygosity regions in AQ pigs are mainly associated with immune responses.^13^ SS is also extremely adaptable to the adverse environment, and can survive in different temperature and humidity environments. The acquisition of candidate genes (*PIK3IP1*, *TUG1*, and *SELENOM*) related to environmental adaptability and disease-resistance of these breeds is of great significance for the development of the pig industry in the wake of climate change. The PI3 kinase (PI3K) pathway is closely related to the activation and development of T cells; *Pik3ip1* has been reported to be a negative regulatory factor for the PI3K pathway, and T cell activation was enhanced by silencing PIK3IP1.^24^ Other studies have highlighted the importance of Pik3ip1 in the treatment of autoimmune diseases.^25^ In addition, *Pik3ip1* was identified as a candidate gene associated with disease resistance in the selected region of Xiang pigs.^26^ Su et al. showed that *TUG1* can protect the cold-storage liver and decrease inflammation and immune response.^27^ The transcription level of *TUG1* gene in the skeletal muscles of hibernating animals was significantly higher than that in endotherms.^28^ The abovementioned results suggest the role of the *TUG1* gene in cold adaptation. In a previous report, the *SELENOM* gene was screened in AQ and Henan native pig breeds in selected regions, and it was associated with growth and fat deposition.^29^^,^^30^ Conversely, broilers are highly sensitive to environmental heat stress because of their fast growth rate and high metabolic rate. A recent study shows that promoting the expression of SELENOM protein can alleviate liver damage and metabolic disorders caused by heat stress, which suggests that *SELENOM* may help organisms adapt to high temperatures through the maintenance of mitochondrial and endoplasmic reticulum homeostasis.^31^

### Breeding objectives for LW pigs from population-specific selective signatures

Reproductive performance is a key animal trait in the pig industry as it directly affects economic output. LW pigs are commonly used as dams in pig breeding systems, the most representative case is as the first-dam in a three-way hybrid pig (Duroc × [Landrace × LW]), and to this end, LW pigs have long been subjected to artificial selection associated with fertility traits. Since 1992, the ability to increase litter size has been one of the main goals of the pig breeding industry in Denmark, and Danish LW pigs are known for their high fecundity in Europe.^32^ Additionally, an introgressive genomic region from a prominent Chinese breed (Taihu Lake pig) has been found in the Danish LW pig genome, which has pig reproductive performance-promotion effects.^33^ In this study, we obtained five candidate genes related to reproductive traits in LW pigs from selected regions: *ALDH1A2*, *APC2*, *PTBP1*, *APQ9*, and *FGF7*. Ovary steroid hormones are extensively involved in reproduction, and dysregulation of ovarian steroid hormone biosynthesis may reduce reproductive performance in sows.^34^ *ALDH1A2* regulates retinoic acid metabolism in granulosa cells to influence progesterone synthesis in ovarian granulosa cells.^35^ At SSC1:113.91–113.95 Mb, the *ALDH1A2* gene is located around the top SNP (SSC1:113925836), which impacts the number of live births from Landrace sows.^36^ Vainios et al. showed that the signaling molecule WNT-4 is essential for the sexual development of the female,^37^ and the WNT pathway is a major signaling pathway of estradiol synthesis in goat ovarian granulosa cells.^38^ In our study, the WNT signaling pathway was significantly enriched in the LW pig group, according to KEGG analysis. As the receptor of WNT, *APC2* mRNA expression is significantly higher when Cu and Cu + Se activate WNT2/4 signaling in caprine ovarian granulosa cells.^39^ Polypyrimidine tract-binding protein (PTBP1) plays a crucial role in alternative splicing of pre-mRNA in eukaryotic cells, and is involved in various cellular processes, including RNA metabolism, gene expression regulation, and cell differentiation.^40^^,^^41^ Suckale et al. reported that the *PTBP1* gene plays an important role in regulating embryonic development in mice, and embryos lacking *PTBP1* show developmental delay or stagnation.^42^ Interestingly, both *APC2* and *PTBP1* genes were also selected in a selection signal study of LW sows with high and low estimated breeding values of total litter size.^43^ Aquaporins (AQPs) are water channel proteins responsible for water homeostasis, and they play a key role in reproductive fluid homeostasis; *AQP9* gene expression is associated with the estrus cycle and pregnancy in the porcine oviduct.^44^^,^^45^ *FGF7* may be an important regulator for bovine early antral follicle growth and its action; *FGF7* can promote the maturation of porcine oocytes *in vitro*, as evidenced by the increase in oocyte diameter, upregulation of anti-apoptosis gene expression, and decrease in reactive oxygen species levels.^46^^,^^47^ In addition, *FGF7* gene was reportedly differentially expressed in the ovaries of high and low egg production Nandanyao hens by RNA-sequencing, and further analysis indicated that the *FGF7* gene is a potential a key gene affecting egg production in Nandanyao hens^48^

## Conclusions

In this study, we obtained genetic variation information of Danish LW pigs and Chinese indigenous pigs based on genome resequencing data, and analyzed genome-wide selection signals of the two groups; 257 and 28 selective sweep regions were identified, respectively. Moreover, we found a series of candidate genes related to reproductive performance and environmental adaptability. The results of this study advance our understanding of artificial and natural selective processes during intense selection and enrich the selectable sites that can advance pig genetic breeding.

## Supplementary Material

Supplementary Materials.pdf

## Data Availability

The WGS datasets of the AQ and SS pigs were submitted to NCBI (National Center for Biotechnology Information, https://www.ncbi.nlm.nih.gov/) under the accession number PRJNA699491. The WGS datasets of LW, DQZ, and DN pigs are available from the corresponding author upon reasonable request.
