# Supplementary material for: Whole-genome selective sweep analysis of Danish Large White and Chinese indigenous pig populations
Source: Anim Biotechnol. 2025 Mar 1;36(1):2467411. doi: 10.1080/10495398.2025.2467411 (PMC12674217; doi:10.1080/10495398.2025.2467411)
Supplement: Supplementary Materials.pdf [file LABT_A_2467411_SM0345.pdf]

Table S1 The information for the selected regions of non-LW group					
Chr	Start	End	Fst	Pi_LW/Pi_none_LW	Genes
1	125950001	126050000	0.641950249	1.211671219	--
1	125960001	126060000	0.636483099	1.183716321	--
5	38720001	38820000	0.620133179	1.184202144	ENSSSCG00000025915;<br>ENSSSCG00000000519;<br>ENSSSCG00000033524
14	47650001	47750000	0.608544656	1.716639443	ENSSSCG00000010015
14	47720001	47820000	0.606317596	1.678608455	ENSSSCG00000010015;<br>ENSSSCG00000010016;<br>ENSSSCG00000057183;<br>ENSSSCG00000040413
14	47730001	47830000	0.608286524	1.61190666	ENSSSCG00000010015;<br>ENSSSCG00000010016;<br>ENSSSCG00000057183;<br>ENSSSCG00000040413
14	47740001	47840000	0.61293163	1.669839029	ENSSSCG00000010016;<br>ENSSSCG00000057183;<br>ENSSSCG00000040413
14	47750001	47850000	0.618172889	1.702365921	ENSSSCG00000010016;<br>ENSSSCG00000057183;<br>ENSSSCG00000040413
14	47760001	47860000	0.625959125	1.771111418	ENSSSCG00000010016;<br>ENSSSCG00000057183;<br>ENSSSCG00000040413
14	47770001	47870000	0.633423759	2.028074673	ENSSSCG00000010016;<br>ENSSSCG00000057183;<br>ENSSSCG00000040413

14	47780001	47880000	0.632222186	2.063055624	ENSSSCG00000010016;<br>ENSSSCG00000057183;<br>ENSSSCG00000040413;<br>ENSSSCG00000010017
14	47790001	47890000	0.630988177	2.027331427	ENSSSCG00000010016;<br>ENSSSCG00000057183;<br>ENSSSCG00000040413;<br>ENSSSCG00000010017;<br>ENSSSCG00000063554
14	47800001	47900000	0.6327614	2.014750559	ENSSSCG00000057183;<br>ENSSSCG00000040413;<br>ENSSSCG00000010017;<br>ENSSSCG00000063554
14	47810001	47910000	0.635411181	2.005337746	ENSSSCG00000040413;<br>ENSSSCG00000010017;<br>ENSSSCG00000063554;<br>ENSSSCG00000029781
14	47820001	47920000	0.63339149	1.99442967	ENSSSCG00000010017;<br>ENSSSCG00000063554;<br>ENSSSCG00000029781;<br>ENSSSCG00000030345
14	47830001	47930000	0.636747913	2.090472391	ENSSSCG00000010017;<br>ENSSSCG00000063554;<br>ENSSSCG00000029781;<br>ENSSSCG00000030345;<br>ENSSSCG00000010023
14	47840001	47940000	0.63574009	1.9613294	ENSSSCG00000010017;<br>ENSSSCG00000063554;<br>ENSSSCG00000029781;<br>ENSSSCG00000030345;<br>ENSSSCG00000010023

14	47850001	47950000	0.644714812	1.913342458	ENSSSCG00000010017; ENSSSCG00000063554; ENSSSCG00000029781; ENSSSCG00000030345; ENSSSCG00000010023
14	47860001	47960000	0.65259301	1.909706992	ENSSSCG00000010017; ENSSSCG00000063554; ENSSSCG00000029781; ENSSSCG00000030345; ENSSSCG00000010023; ENSSSCG00000058877
14	47870001	47970000	0.659695648	1.697483286	ENSSSCG00000010017; ENSSSCG00000063554; ENSSSCG00000029781; ENSSSCG00000030345; ENSSSCG00000010023; ENSSSCG00000058877
14	47880001	47980000	0.663865155	1.785922649	ENSSSCG00000010017; ENSSSCG00000063554; ENSSSCG00000029781; ENSSSCG00000030345; ENSSSCG00000010023; ENSSSCG00000058877
14	47890001	47990000	0.65731975	1.741562064	ENSSSCG00000010017; ENSSSCG00000029781; ENSSSCG00000030345; ENSSSCG00000010023; ENSSSCG00000058877;

14	47900001	48000000	0.65586305	1.719101574	ENSSSCG00000010017; ENSSSCG00000029781; ENSSSCG00000030345; ENSSSCG00000010023; ENSSSCG00000058877; ENSSSCG00000010025
14	47910001	48010000	0.645678785	1.630110779	ENSSSCG00000030345; ENSSSCG00000010023; ENSSSCG00000058877; ENSSSCG00000010025
14	47920001	48020000	0.639134205	1.578457993	ENSSSCG00000030345; ENSSSCG00000010023; ENSSSCG00000058877; ENSSSCG00000010025
14	47930001	48030000	0.633639603	1.54476878	ENSSSCG00000058877; ENSSSCG00000010025
14	47940001	48040000	0.624980314	1.519418766	ENSSSCG00000058877; ENSSSCG00000010025
14	47950001	48050000	0.608165875	1.432681756	ENSSSCG00000058877; ENSSSCG00000010025; ENSSSCG00000010026

Table S2 The information for the selected regions of LW group					
Chr	Start	End	Fst	Pi_none_LW/Pi_LW	Genes
1	113810001	113910000	0.719377643	80.47058578	ENSSSCG00000004597;ENSSSCG00000046774
1	113820001	113920000	0.730042571	89.58951544	ENSSSCG00000004597;ENSSSCG00000046774
1	113830001	113930000	0.743416364	82.27616037	ENSSSCG00000004597;ENSSSCG00000046774
1	113840001	113940000	0.76030333	73.61072969	ENSSSCG00000004597;ENSSSCG00000046774;ENSSSCG00000025578
1	113850001	113950000	0.768254679	70.15326818	ENSSSCG00000046774;ENSSSCG00000025578
1	115630001	115730000	0.646510896	71.3332135	ENSSSCG00000057596
1	115640001	115740000	0.663746029	77.69470856	ENSSSCG00000057596;ENSSSCG00000004603
1	120290001	120390000	0.607790368	103.7892423	ENSSSCG00000032517;ENSSSCG00000004632
1	120310001	120410000	0.605592563	80.04623961	ENSSSCG00000004632

1	120320001	120420000	0.606889293	74.78155606	ENSSSCG00000004632
1	120470001	120570000	0.643856062	70.39109497	ENSSSCG00000030168
1	122220001	122320000	0.619567225	99.42515647	ENSSSCG00000004648;ENSSSCG00000056997
1	122230001	122330000	0.63922011	120.3025015	ENSSSCG00000004648;ENSSSCG00000056997
1	122240001	122340000	0.638149831	107.4396359	ENSSSCG00000004648;ENSSSCG00000056997
1	122250001	122350000	0.635281935	111.1573821	ENSSSCG00000004648;ENSSSCG00000056997
1	122260001	122360000	0.634247266	101.7119308	ENSSSCG00000004648;ENSSSCG00000056997
1	122270001	122370000	0.634804471	83.47526636	ENSSSCG00000004648;ENSSSCG00000056997;ENSSSCG00000004651
1	135740001	135840000	0.687438502	76.96941965	ENSSSCG00000035254;ENSSSCG00000054956
1	135750001	135850000	0.650739885	92.36937909	ENSSSCG00000035254;ENSSSCG00000054956

1	135760001	135860000	0.636577102	110.7163045	ENSSSCG00000054956
1	135770001	135870000	0.613966729	127.5532097	ENSSSCG00000054956
1	142390001	142490000	0.687340641	67.08955675	ENSSSCG00000004834;ENSSSCG00000055131;ENSSSCG00000061428
1	142450001	142550000	0.662934096	66.20864003	ENSSSCG00000055131;ENSSSCG00000061428;ENSSSCG00000004836;ENSSSCG00000056355
1	142640001	142740000	0.614488853	94.87999235	--
1	142650001	142750000	0.610187566	111.398107	--
1	142660001	142760000	0.612495095	121.3232142	--
1	142670001	142770000	0.628618864	94.55071202	--
1	142680001	142780000	0.62568046	81.8547153	--
1	142690001	142790000	0.610128016	100.2818796	--
1	142700001	142800000	0.610322781	135.250699	ENSSSCG00000053349
1	142710001	142810000	0.643062107	77.49930997	ENSSSCG00000053349;ENSSSCG00000004839
1	142720001	142820000	0.638091944	75.80737231	ENSSSCG00000053349;ENSSSCG00000004839

1	142730001	142830000	0.624253846	80.82424484	ENSSSCG00000053349;ENSSSCG00000004839
1	143440001	143540000	0.60687441	82.15616359	ENSSSCG00000053952;ENSSSCG00000026006
1	143450001	143550000	0.646732893	79.17793942	ENSSSCG00000053952;ENSSSCG00000026006
1	143460001	143560000	0.648552097	93.57538671	ENSSSCG00000026006
1	143470001	143570000	0.612293081	75.24809149	ENSSSCG00000026006;ENSSSCG00000058025
1	143490001	143590000	0.606598726	70.23032491	ENSSSCG00000026006;ENSSSCG00000058025
1	145900001	146000000	0.612561105	91.73803546	ENSSSCG00000004856
1	227390001	227490000	0.612852124	70.32816643	--
2	48880001	48980000	0.608057026	71.95145603	ENSSSCG00000020720;ENSSSCG00000029886
2	50240001	50340000	0.646896457	66.75212665	--
2	50250001	50350000	0.619805194	66.06000526	--
2	54580001	54680000	0.611052261	78.00348397	ENSSSCG00000062218;ENSSSCG00000052160

2	59700001	59800000	0.61304029	161.800497	ENSSSCG00000013894;ENSSSCG00000058229;ENSSSCG00000013893;ENSSSCG00000013892
2	59710001	59810000	0.639614643	103.9627982	ENSSSCG00000013894;ENSSSCG00000058229;ENSSSCG00000013893;ENSSSCG00000013892
2	59720001	59820000	0.656519832	91.0164952	ENSSSCG00000013894;ENSSSCG00000058229;ENSSSCG00000013893;ENSSSCG00000013892
2	59730001	59830000	0.677217353	80.37576415	ENSSSCG00000013894;ENSSSCG00000058229;ENSSSCG00000013893;ENSSSCG00000013892;ENSSSCG00000013891
2	59740001	59840000	0.701206759	80.19775251	ENSSSCG00000058229;ENSSSCG00000013893;ENSSSCG00000013892;ENSSSCG00000013891

2	59750001	59850000	0.69474487	88.49101115	ENSSSCG00000058229;ENSSSCG00000013893;ENSSSCG00000013892;ENSSSCG00000013891
2	59760001	59860000	0.677189454	119.1683879	ENSSSCG00000013893;ENSSSCG00000013892;ENSSSCG00000013891
2	59770001	59870000	0.654223434	109.6364453	ENSSSCG00000013893;ENSSSCG00000013892;ENSSSCG00000013891;ENSSSCG00000013890
2	72220001	72320000	0.683522747	66.86742387	ENSSSCG00000013556
2	72230001	72330000	0.682579967	79.51571615	ENSSSCG00000013556;ENSSSCG00000013553
2	72240001	72340000	0.679689003	119.3837719	ENSSSCG00000013556;ENSSSCG00000013553
2	72250001	72350000	0.668646771	147.1966349	ENSSSCG00000013556;ENSSSCG00000013553
2	72260001	72360000	0.645831666	206.224831	ENSSSCG00000013556;ENSSSCG00000013553

2	72270001	72370000	0.64213836	145.0802291	ENSSSCG00000013556;ENSSSCG00000013553
2	72280001	72380000	0.63640768	154.3517621	ENSSSCG00000013556;ENSSSCG00000013553
2	72290001	72390000	0.633127609	159.7255116	ENSSSCG00000013556;ENSSSCG00000013553
2	72300001	72400000	0.616206577	168.6531546	ENSSSCG00000013556;ENSSSCG00000013553;ENSSSCG00000022380
2	72310001	72410000	0.616915029	155.331867	ENSSSCG00000013553;ENSSSCG00000022380;ENSSSCG00000013554
2	73130001	73230000	0.629759721	409.3090449	ENSSSCG00000031764;ENSSSCG00000027307;ENSSSCG00000013530;ENSSSCG00000033413;ENSSSCG00000040968;ENSSSCG00000013527
2	73140001	73240000	0.635704449	461.0967034	ENSSSCG00000013530;ENSSSCG00000033413;ENSSSCG00000040968;ENSSSCG00000013527

2	73150001	73250000	0.635968305	543.8394861	ENSSSCG00000013530;ENSSSCG00000033413;ENSSSCG00000040968;ENSSSCG00000013527;ENSSSCG00000061996
2	73160001	73260000	0.622815994	581.0549377	ENSSSCG00000013530;ENSSSCG00000033413;ENSSSCG00000040968;ENSSSCG00000013527;ENSSSCG00000061996
2	73170001	73270000	0.613753964	1139.508888	ENSSSCG00000013530;ENSSSCG00000033413;ENSSSCG00000040968;ENSSSCG00000013527;ENSSSCG00000061996;ENSSSCG00000027128
2	73180001	73280000	0.607346656	1179.335807	ENSSSCG00000033413;ENSSSCG00000040968;ENSSSCG00000013527;ENSSSCG00000061996;ENSSSCG00000027128
2	73540001	73640000	0.665580554	68.80090338	ENSSSCG00000028612
2	73550001	73650000	0.6780061	73.19445492	ENSSSCG00000028612

2	73560001	73660000	0.692838382	71.67194883	ENSSSCG00000028612
2	73570001	73670000	0.699686346	79.66597048	ENSSSCG00000028612
2	73580001	73680000	0.704155831	99.71564884	ENSSSCG00000028612
2	73590001	73690000	0.675413418	109.8416829	ENSSSCG00000028612
2	73600001	73700000	0.646324605	225.706199	ENSSSCG00000028612
2	73610001	73710000	0.624058315	88.52616295	ENSSSCG00000028612
2	75410001	75510000	0.621516844	84.29408979	ENSSSCG00000013473;ENSSSCG00000029279;ENSSSCG00000022205;ENSSSCG00000032265
2	75420001	75520000	0.63492148	77.28484823	ENSSSCG00000013473;ENSSSCG00000029279;ENSSSCG00000022205;ENSSSCG00000032265;ENSSSCG00000028995
2	75430001	75530000	0.640991642	105.1733011	ENSSSCG00000013473;ENSSSCG00000029279;ENSSSCG00000022205;ENSSSCG00000032265;ENSSSCG00000028995

2	75440001	75540000	0.641910894	105.6433305	ENSSSCG00000029279;ENSSSCG00000022205;ENSSSCG00000032265;ENSSSCG00000028995;ENSSSCG00000024588
2	75450001	75550000	0.635884722	109.0629436	ENSSSCG00000029279;ENSSSCG00000022205;ENSSSCG00000032265;ENSSSCG00000028995;ENSSSCG00000024588;ENSSSCG00000053447
2	75460001	75560000	0.616701586	104.0504674	ENSSSCG00000022205;ENSSSCG00000032265;ENSSSCG00000028995;ENSSSCG00000024588;ENSSSCG00000053447
2	75610001	75710000	0.641918192	66.85013325	ENSSSCG00000036792;ENSSSCG00000059026;ENSSSCG00000034545
2	75620001	75720000	0.679944604	69.03967374	ENSSSCG00000036792;ENSSSCG00000059026;ENSSSCG00000034545

2	75630001	75730000	0.70237687	67.53538061	ENSSSCG00000059026;ENSSSCG00000034545
2	75650001	75750000	0.715451804	76.89771973	ENSSSCG00000059026;ENSSSCG00000034545;ENSSSCG00000013471
2	75660001	75760000	0.717484842	70.42576057	ENSSSCG00000034545;ENSSSCG00000013471
2	76660001	76760000	0.620501628	117.5765159	ENSSSCG00000013442;ENSSSCG00000024672;ENSSSCG00000040624;ENSSSCG00000013440;ENSSSCG00000013439
2	76670001	76770000	0.619713309	109.0842105	ENSSSCG00000024672;ENSSSCG00000040624;ENSSSCG00000013440;ENSSSCG00000013439
2	76680001	76780000	0.609475763	134.1204119	ENSSSCG00000024672;ENSSSCG00000040624;ENSSSCG00000013440;ENSSSCG00000013439;ENSSSCG00000049245

2	76970001	77070000	0.605842951	117.1105486	ENSSSCG00000013433;ENSSSCG00000026387;ENSSSCG00000024379;ENSSSCG00000022265;ENSSSCG00000023329;ENSSSCG00000033169;ENSSSCG00000013427
2	76990001	77090000	0.617855963	89.72580795	ENSSSCG00000024379;ENSSSCG00000022265;ENSSSCG00000023329;ENSSSCG00000033169;ENSSSCG00000013427;ENSSSCG00000023142;ENSSSCG00000024144;ENSSSCG00000013426
2	77530001	77630000	0.610510929	203.8745977	ENSSSCG00000013415;ENSSSCG00000037432;ENSSSCG00000013421;ENSSSCG00000042865;ENSSSCG00000013425;ENSSSCG00000039575

2	139880001	139980000	0.613176284	80.2578569	ENSSSCG00000014321;ENSSSCG00000014324;ENSSSCG00000026421;ENSSSCG00000035776;ENSSSCG00000029125
2	142220001	142320000	0.615888553	278.6453599	ENSSSCG00000014364;ENSSSCG00000019080;ENSSSCG00000059551;ENSSSCG00000055768;ENSSSCG00000014366
4	51790001	51890000	0.612685765	95.23205552	ENSSSCG00000006149
4	51800001	51900000	0.629515438	93.19386497	ENSSSCG00000006149
4	51810001	51910000	0.625130902	97.29242838	ENSSSCG00000006149
4	51820001	51920000	0.625377015	73.35676757	ENSSSCG00000006149
4	51830001	51930000	0.618824498	75.60071712	ENSSSCG00000006149
4	51840001	51940000	0.616978572	73.1170475	ENSSSCG00000006149
4	52090001	52190000	0.645562533	75.01708539	ENSSSCG00000006149;ENSSSCG00000057461

4	52100001	52200000	0.665444049	101.0892932	ENSSSCG00000006149;ENSSSCG00000057461
4	52110001	52210000	0.670600266	137.0980043	ENSSSCG00000006149;ENSSSCG00000057461
4	52120001	52220000	0.669869838	139.3193854	ENSSSCG00000006149;ENSSSCG00000057461
4	52130001	52230000	0.664627826	92.8582868	ENSSSCG00000006149;ENSSSCG00000057461
4	52140001	52240000	0.64621491	133.8807094	ENSSSCG00000006149;ENSSSCG00000057461
4	52150001	52250000	0.62991849	100.8584391	ENSSSCG00000006149
4	52160001	52260000	0.614062032	101.7495174	ENSSSCG00000006149
4	52380001	52480000	0.607300505	93.09746585	ENSSSCG00000006149;ENSSSCG00000019766
4	52690001	52790000	0.609974098	80.66028693	--
4	52700001	52800000	0.627322525	74.1636581	--
4	52710001	52810000	0.611410456	78.5600289	--
4	52720001	52820000	0.611218934	73.46535472	--
4	52740001	52840000	0.606383133	73.85318024	--

5	82250001	82350000	0.633272277	85.65331465	ENSSSCG00000023972;ENSSSCG00000056574
5	82260001	82360000	0.647050948	102.9228751	ENSSSCG00000023972;ENSSSCG00000056574
5	82270001	82370000	0.645799841	102.6262715	ENSSSCG00000023972;ENSSSCG00000056574;ENSSSCG00000000862
5	82280001	82380000	0.638977105	81.73344689	ENSSSCG00000023972;ENSSSCG00000056574;ENSSSCG00000000862
5	82290001	82390000	0.639283035	76.58082151	ENSSSCG00000023972;ENSSSCG00000056574;ENSSSCG00000000862
5	82300001	82400000	0.629323486	81.11807517	ENSSSCG00000023972;ENSSSCG00000056574;ENSSSCG00000000862
5	82310001	82410000	0.633466226	77.7403084	ENSSSCG00000023972;ENSSSCG00000056574;ENSSSCG00000000862

5	82320001	82420000	0.629397508	88.56054028	ENSSSCG00000023972;ENSSSCG00000056574;ENSSSCG00000000862
5	82330001	82430000	0.61706306	95.08504338	ENSSSCG00000000862
7	103400001	103500000	0.619671447	294.1421308	ENSSSCG00000002410
7	103410001	103510000	0.610119069	238.831744	ENSSSCG00000002410
8	12820001	12920000	0.622090988	317.7216504	ENSSSCG00000008748
8	12830001	12930000	0.643219886	288.9464778	ENSSSCG00000008748
8	12840001	12940000	0.648937502	295.0065117	ENSSSCG00000008748
8	12850001	12950000	0.653644564	285.4156776	ENSSSCG00000008748
8	12860001	12960000	0.654835002	300.1667207	ENSSSCG00000008748
8	12870001	12970000	0.657703803	299.1490564	ENSSSCG00000008748
8	12880001	12980000	0.615925738	227.0244233	ENSSSCG00000008748
8	46290001	46390000	0.662041612	66.95970567	--
8	46300001	46400000	0.668207246	80.06558026	--
8	46310001	46410000	0.67708934	70.29865715	--
8	46320001	46420000	0.676890011	89.17400034	--
8	46330001	46430000	0.671922052	96.04710583	--

8	46700001	46800000	0.637748654	112.2555078	ENSSSCG00000053870
8	46710001	46810000	0.679383781	88.20266617	ENSSSCG00000053870
8	46770001	46870000	0.803757978	72.75025938	ENSSSCG00000039268
8	46780001	46880000	0.817008998	76.84280459	ENSSSCG00000039268
8	46810001	46910000	0.725492776	72.13421043	ENSSSCG00000039268
8	46820001	46920000	0.687734993	76.59785654	ENSSSCG00000039268
8	46840001	46940000	0.644440721	68.43466296	ENSSSCG00000039268
8	47200001	47300000	0.620587895	80.77856943	ENSSSCG00000008874;ENSSSCG00000041883
8	47210001	47310000	0.660040969	84.34725854	ENSSSCG00000008874
8	47220001	47320000	0.663434914	91.94688565	--
8	47230001	47330000	0.651895036	105.8380783	--
8	47240001	47340000	0.663973683	144.35327	--
8	47250001	47350000	0.664788745	133.4987587	--
8	47260001	47360000	0.653983619	79.72506439	--
8	47270001	47370000	0.667609973	66.1085324	--
8	47290001	47390000	0.62568663	72.80955202	--
8	47440001	47540000	0.614962151	248.1291025	ENSSSCG00000008875

8	47450001	47550000	0.643251032	234.7337158	ENSSSCG00000008875
8	47460001	47560000	0.660358781	240.3403568	ENSSSCG00000008875
8	47470001	47570000	0.685662371	108.3113356	ENSSSCG00000008875
8	47480001	47580000	0.699506728	68.66093705	ENSSSCG00000008875
8	47590001	47690000	0.6168529	113.8116321	ENSSSCG00000008875;ENSSSCG00000028492;ENSSSCG00000008877;ENSSSCG00000008878
8	47600001	47700000	0.607848321	108.3582048	ENSSSCG00000008875;ENSSSCG00000028492;ENSSSCG00000008877;ENSSSCG00000008878
8	48370001	48470000	0.651916768	67.71767346	--
8	48380001	48480000	0.654531355	96.59795166	--
8	48390001	48490000	0.645562183	83.70812454	--
8	48400001	48500000	0.64340778	87.95005238	--
8	48410001	48510000	0.658222229	87.14089684	--
8	48420001	48520000	0.662751716	125.1204417	ENSSSCG00000057421
8	48430001	48530000	0.659882125	133.2669295	ENSSSCG00000057421
8	48440001	48540000	0.655303702	135.7659835	ENSSSCG00000057421

8	48450001	48550000	0.652207056	188.0686961	ENSSSCG00000057421
8	48460001	48560000	0.652660936	151.236379	ENSSSCG00000057421
8	48470001	48570000	0.648658226	145.5599833	ENSSSCG00000057421
8	48480001	48580000	0.652605017	141.9509212	ENSSSCG00000057421
8	48490001	48590000	0.660660805	188.7451627	ENSSSCG00000057421
8	48500001	48600000	0.646216444	122.1713634	ENSSSCG00000057421
8	48510001	48610000	0.6265099	100.1374062	ENSSSCG00000057421
8	48520001	48620000	0.639153458	88.77975433	--
8	48530001	48630000	0.650101414	108.0450813	--
8	48540001	48640000	0.676636777	99.15595449	ENSSSCG00000062953
8	48550001	48650000	0.691018148	66.87870243	ENSSSCG00000062953
8	48560001	48660000	0.69885801	79.54240626	ENSSSCG00000062953
8	48570001	48670000	0.696150789	80.05347558	ENSSSCG00000062953
8	48580001	48680000	0.676812531	83.36697676	ENSSSCG00000062953
8	48690001	48790000	0.645335161	70.46992977	--
8	48700001	48800000	0.624667972	104.5101958	--
8	48710001	48810000	0.618735009	104.5436091	--

8	48950001	49050000	0.755846377	68.37144611	--
8	48960001	49060000	0.739024677	68.86062902	--
8	48970001	49070000	0.72271783	72.74790744	--
8	49100001	49200000	0.640661578	190.9514447	--
8	49110001	49210000	0.630784444	113.627638	--
8	49120001	49220000	0.63084085	109.5772108	--
8	49130001	49230000	0.632402309	79.96858199	--
8	49240001	49340000	0.829849682	71.26390298	--
8	49420001	49520000	0.606622345	69.23061008	--
8	49720001	49820000	0.606529737	84.39777794	--
8	49730001	49830000	0.643102823	115.5395823	--
8	49740001	49840000	0.676348179	184.0327803	--
8	49750001	49850000	0.67544611	185.7273272	--
8	49760001	49860000	0.673221345	188.3343626	--
8	49770001	49870000	0.67659467	2495.167124	--
8	49780001	49880000	0.683655525	121.3014954	--
8	49790001	49890000	0.689962343	126.3071182	--
8	49800001	49900000	0.693066944	78.77173066	--
8	49810001	49910000	0.711248635	73.2506373	--
8	49880001	49980000	0.79478704	91.43358007	--
8	49890001	49990000	0.775829424	71.83564744	--
8	49900001	50000000	0.759542932	79.38604827	--
8	49910001	50010000	0.722727773	71.46252232	--
8	49920001	50020000	0.681743466	76.41346797	--
8	49930001	50030000	0.629534943	70.2067661	--
8	50220001	50320000	0.618829942	73.92137237	ENSSSCG00000027183
8	50230001	50330000	0.610432691	107.6306698	ENSSSCG00000027183

8	50430001	50530000	0.613587806	120.0707052	ENSSSCG00000027183
8	50440001	50540000	0.64676307	77.12150864	ENSSSCG00000027183
8	50450001	50550000	0.678202191	79.3606372	ENSSSCG00000027183
8	50460001	50560000	0.699468565	75.335994	ENSSSCG00000027183
8	50490001	50590000	0.730090733	77.70365645	ENSSSCG00000027183
8	50520001	50620000	0.687887045	86.67366742	ENSSSCG00000027183
8	50530001	50630000	0.665250714	92.05632921	ENSSSCG00000027183
8	50540001	50640000	0.621613754	150.9912917	ENSSSCG00000027183
8	50800001	50900000	0.624972153	67.65576732	ENSSSCG00000027183
8	50810001	50910000	0.679910018	89.31534638	ENSSSCG00000027183
8	50820001	50920000	0.728861517	121.7330255	ENSSSCG00000027183
8	50830001	50930000	0.723354592	105.9276583	ENSSSCG00000027183
8	50840001	50940000	0.709594002	98.80782094	ENSSSCG00000027183
8	50850001	50950000	0.707070191	109.9278476	ENSSSCG00000027183

8	50860001	50960000	0.725934313	133.4975082	ENSSSCG00000027183
8	50870001	50970000	0.730847004	195.2554828	ENSSSCG00000027183
8	50880001	50980000	0.730158763	198.5461418	ENSSSCG00000027183
8	50890001	50990000	0.727792211	259.7873183	ENSSSCG00000027183
8	50900001	51000000	0.724157557	249.8446437	ENSSSCG00000027183
8	50910001	51010000	0.72469187	228.0496045	ENSSSCG00000027183
8	50920001	51020000	0.705252202	126.9819975	ENSSSCG00000027183
8	50930001	51030000	0.723835191	145.1752206	ENSSSCG00000027183
8	50940001	51040000	0.758485929	90.74486317	ENSSSCG00000027183
8	51040001	51140000	0.718615534	74.58622886	ENSSSCG00000027183;ENSSSCG00000060187;ENSSSCG00000057733
8	51050001	51150000	0.681474905	120.7321606	ENSSSCG00000027183;ENSSSCG00000060187;ENSSSCG00000057733
8	51060001	51160000	0.656574414	80.70902171	ENSSSCG00000060187;ENSSSCG00000057733

8	51070001	51170000	0.639251484	81.86545406	ENSSSCG00000057733
8	51080001	51180000	0.63989524	116.0521333	ENSSSCG00000057733
8	51090001	51190000	0.638193106	116.8955764	ENSSSCG00000057733
8	51100001	51200000	0.646863056	109.6124712	ENSSSCG00000057733
8	51230001	51330000	0.621974568	338.486528	ENSSSCG00000052750
8	51240001	51340000	0.623671418	303.9272273	ENSSSCG00000052750
8	51250001	51350000	0.635659223	134.1599325	ENSSSCG00000052750;ENSSSCG00000018686
8	51260001	51360000	0.632666956	184.707272	ENSSSCG00000052750;ENSSSCG00000018686
8	51270001	51370000	0.622948067	83.30536922	ENSSSCG00000052750;ENSSSCG00000018686
8	51280001	51380000	0.635754636	73.07098505	ENSSSCG00000052750;ENSSSCG00000018686
8	51290001	51390000	0.637992512	69.32436433	ENSSSCG00000052750;ENSSSCG00000018686
8	51580001	51680000	0.725644895	125.6620765	ENSSSCG00000060672

8	51590001	51690000	0.692486233	89.10639792	ENSSSCG00000060672
8	51600001	51700000	0.660083715	96.03588377	ENSSSCG00000060672
8	51610001	51710000	0.618880239	88.42286103	ENSSSCG00000060672
16	48500001	48600000	0.612585078	143.5716836	ENSSSCG00000023818;ENSSSCG00000046085
16	48510001	48610000	0.615056845	147.7387257	ENSSSCG00000023818;ENSSSCG00000046085;ENSSSCG00000016975
16	48520001	48620000	0.61104261	108.7160741	ENSSSCG00000023818;ENSSSCG00000046085;ENSSSCG00000016975

Table S3 GO analysis of candidate genes of non-LW pigs（significantly enriched terms）				
id	num	qvalue	class	Descrption
GO:0034485	1	0.029555759	Molecular Function	phosphatidylinositol-3,4,5-trisphosphate 5-phosphatase
GO:0036313	1	0.029555759	Molecular Function	phosphatidylinositol 3-kinase catalytic subunit binding
GO:0052659	1	0.029555759	Molecular Function	inositol-1,3,4,5-tetrakisphosphate 5-
GO:0052743	1	0.029555759	Molecular Function	inositol tetrakisphosphate phosphatase activity
GO:0004445	1	0.029555759	Molecular Function	inositol-polyphosphate 5-phosphatase activity
GO:0034594	1	0.029555759	Molecular Function	phosphatidylinositol trisphosphate phosphatase
GO:0052658	1	0.029555759	Molecular Function	inositol-1,4,5-trisphosphate 5-phosphatase activity
GO:0046030	1	0.032317957	Molecular Function	inositol trisphosphate

Table S4 GO analysis of candidate genes of LW pigs（significantly enriched terms）				
id	num	qvalue	class	Descrption
GO:0007212	4	0.01184412	Biological Process	dopamine receptor signaling pathway
GO:1903350	4	0.023894656	Biological Process	response to dopamine
GO:1903351	4	0.023894656	Biological Process	cellular response to dopamine
GO:0071867	4	0.023894656	Biological Process	response to monoamine
GO:0071868	4	0.023894656	Biological Process	cellular response to monoamine stimulus
GO:0071869	4	0.023894656	Biological Process	response to catecholamine
GO:0071870	4	0.023894656	Biological Process	cellular response to catecholamine stimulus

Table S5 The information for the selected regions overlapping QTL database of non-LW group							
Chr	Start	End	trait_name	trait_class	trait_classii	QTL_IDs	overlap_QTL_counts
5	38720001	38820000	Cortisol level	health traits	blood_parameters	24133;24134;24135;24136	4
5	38720001	38820000	Feed conversion ratio	production traits	feed conversion	221075;221076	2
5	38720001	38820000	Subcutaneous fat thickness	meat and carcass traits	fatness	261719;261720;261721;261722	4
14	47650001	48050000	Age at puberty	reprodction traits	reproductive traits	22109	1
14	47650001	48050000	Fat androstenone level	meat and carcass traits	chemistry	194688	1
14	47650001	48050000	Gestation length	reprodction traits	reproductive traits	173177	1
14	47650001	48050000	Mean platelet volume	health traits	blood_parameters	37844	1
14	47650001	48050000	Melanoma susceptibility	health traits	disease susceptibility	170948	1
14	47650001	48050000	Teat number	reprodction traits	reproductive	126628	1
14	47650001	48050000	Umbilical hernia	exterior traits	defects	156617;156619;156620;156621;156623;156624;156625	7
14	47650001	48050000	White blood cell number	health traits	immune capacity	237793;237794;237795;237796;237797;237798	6

Table S6 The information for the selected regions overlapping QTL database of LW group							
Chr	Start	End	trait_name	trait_class	trait_classii	QTL_IDs	overlap_QTL_counts
2	75410001	75560000	Average backfat thickness	meat and carcass traits	fatness	170625	1
5	82250001	82430000	Average backfat thickness	meat and carcass traits	fatness	9881	1
16	48500001	48620000	Average backfat thickness	meat and carcass traits	fatness	22286	1
5	82250001	82430000	Average daily gain	production traits	growth	9886	1
2	73130001	73280000	Average glycolytic potential	meat and carcass traits	chemistry	9814	1
2	73540001	73710000	Average glycolytic potential	meat and carcass traits	chemistry	9814	1
5	82250001	82430000	Average glycolytic potential	meat and carcass traits	chemistry	9894	1
2	73130001	73280000	Average lactate	meat and carcass traits	chemistry	9815	1
2	73540001	73710000	Average lactate	meat and carcass traits	chemistry	9815	1
5	82250001	82430000	Average lactate	meat and carcass traits	chemistry	9895	1
5	82250001	82430000	Backfat at last lumbar	meat and carcass traits	fatness	9883	1
5	82250001	82430000	Backfat at last rib	meat and carcass traits	fatness	9882	1
5	82250001	82430000	Backfat at tenth rib	meat and carcass traits	fatness	9884	1
1	227390001	227490000	Body weight	production traits	growth	263730	1
5	82250001	82430000	Body weight	production traits	growth	9885	1
5	82250001	82430000	Carcass weight (hot)	meat and carcass traits	anatomy	9880	1

5	82250001	82430000	Cooking loss	meat and carcass traits	texture	9891	1
2	139880001	139980000	Corpus luteum number	reprodction traits	litter traits	31837	1
2	77530001	77630000	Cross-sectional area of type IIa muscle fibers	meat and carcass traits	anatomy	10238	1
1	120290001	120420000	Drip loss	meat and carcass traits	texture	7744;7826;7949;8350	4
1	120470001	120570000	Drip loss	meat and carcass traits	texture	7744;7826;7949;8350	4
1	142390001	142550000	Drip loss	meat and carcass traits	texture	7993;8121;8380;8444;8518	5
1	142640001	142830000	Drip loss	meat and carcass traits	texture	7993;8121;8380;8444;8518	5
1	227390001	227490000	Fat to meat ratio	meat and carcass traits	anatomy	12738	1
2	73130001	73280000	Firmness	meat and carcass traits	texture	9808	1
2	73540001	73710000	Firmness	meat and carcass traits	texture	9808	1
2	73130001	73280000	Flavor score	meat and carcass traits	flavor	9813	1
2	73540001	73710000	Flavor score	meat and carcass traits	flavor	9813	1
2	48880001	48980000	Gestation length	reprodction traits	reproductive traits	18141	1
2	142220001	142320000	Gestation length	reprodction traits	reproductive traits	65331	1
2	75610001	75760000	HDL/LDL ratio	health traits	blood_parameters	23608	1
4	52690001	52840000	Head weight	meat and carcass traits	anatomy	153182	1
8	47590001	47700000	Iris pigmentation	exterior traits	eye color	213042;213088	2
2	76660001	76780000	Juiciness score	meat and carcass traits	flavor	5758	1

2	59700001	59870000	LDL cholesterol	health traits	blood_parameters	23495;23540	2
2	75410001	75560000	LDL cholesterol	health traits	blood_parameters	23575;23576	2
2	75610001	75760000	LDL cholesterol	health traits	blood_parameters	23577	1
2	75410001	75560000	Lean meat percentage	meat and carcass traits	anatomy	170645	1
2	77530001	77630000	Lean meat percentage	meat and carcass traits	anatomy	216174	1
2	76660001	76780000	Longissimus muscle weight	meat and carcass traits	anatomy	6452	1
1	120290001	120420000	Lymphocyte number	health traits	immune capacity	107353	1
8	47590001	47700000	Mean corpuscular hemoglobin concentration	health traits	blood_parameters	22138	1
2	59700001	59870000	Mean corpuscular hemoglobin content	health traits	blood_parameters	27651;27652	2
2	72220001	72410000	Mean corpuscular volume	health traits	blood_parameters	27411	1
8	46700001	46940000	Mean corpuscular volume	health traits	blood_parameters	21473;21474	2
8	47590001	47700000	Mean corpuscular volume	health traits	blood_parameters	22140;22144	2
8	48690001	48810000	Mean corpuscular volume	health traits	blood_parameters	21477;21478;21479	3
8	49240001	49340000	Mean corpuscular volume	health traits	blood_parameters	21481	1
8	49720001	50030000	Mean corpuscular volume	health traits	blood_parameters	21482;21483	2
8	51230001	51390000	Mean corpuscular volume	health traits	blood_parameters	21486	1
2	73130001	73280000	Meat color	meat and carcass traits	meat/fat color	9812	1

2	73540001	73710000	Meat color	meat and carcass traits	meat/fat color	9812	1
5	82250001	82430000	Meat color	meat and carcass traits	meat/fat color	9896;9897	2
2	76970001	77090000	Monocyte number	health traits	immune capacity	5495	1
8	46700001	46940000	Muscle conductivity	meat and carcass traits	conductivity & impedance	161202;161229	2
8	48370001	48680000	Muscle conductivity	meat and carcass traits	conductivity & impedance	160947;160948	2
8	48690001	48810000	Muscle conductivity	meat and carcass traits	conductivity & impedance	160945	1
8	49240001	49340000	Muscle conductivity	meat and carcass traits	conductivity & impedance	160944	1
8	49720001	50030000	Muscle conductivity	meat and carcass traits	conductivity & impedance	160943	1
2	73130001	73280000	Muscle pH	meat and carcass traits	ph	9809	1
2	73540001	73710000	Muscle pH	meat and carcass traits	ph	9809	1
2	75410001	75560000	Number of mummified pigs	reprodction traits	litter traits	18242	1
2	139880001	139980000	Number of mummified pigs	reprodction traits	litter traits	18244	1
4	51790001	51940000	Number of mummified pigs	reprodction traits	litter traits	18299	1
4	52090001	52260000	Number of mummified pigs	reprodction traits	litter traits	18299	1
7	103400001	103510000	Number of ribs	meat and carcass traits	anatomy	160151	1
2	73130001	73280000	Off-flavor score	meat and carcass traits	flavor	9810	1
2	73540001	73710000	Off-flavor score	meat and carcass traits	flavor	9810	1

2	75410001	75560000	Palmitoleic acid content	meat and carcass traits	fatty acid content	176623	1
2	76970001	77090000	Parasite load	health traits	pathogens and parasites	7486	1
8	47440001	47580000	Red blood cell count	health traits	blood_parameters	22145;22165;165079	3
8	47590001	47700000	Red blood cell count	health traits	blood_parameters	22147	1
2	73130001	73280000	Residual feed intake	production traits	feed intake	258118;258119;258120;258121	4
16	48500001	48620000	Rump width	production traits	growth	223618	1
2	76970001	77090000	Scrotal/inguinal hernia	exterior traits	defects	3039	1
2	142220001	142320000	Shear force	meat and carcass traits	texture	5555	1
1	120290001	120420000	Shoulder weight	meat and carcass traits	anatomy	153579	1
1	135740001	135870000	Sperm abnormality rate	reprodction traits	reproductive traits	160625	1
1	135740001	135870000	Sperm motility	reprodction traits	reproductive traits	160623	1
1	135740001	135870000	Sperm progressive motility	reprodction traits	reproductive traits	160624	1
2	77530001	77630000	Subcutaneous fat thickness	meat and carcass traits	fatness	747	1
8	49240001	49340000	Teat number	reprodction traits	reproductive organs	95539	1
8	51040001	51200000	Teat number	reprodction traits	reproductive organs	95540;258413	2
8	47440001	47580000	Tuberculosis susceptibility	health traits	disease susceptibility	160594	1
2	73130001	73280000	Water holding capacity	meat and carcass traits	texture	9811	1
2	73540001	73710000	Water holding capacity	meat and carcass traits	texture	9811	1

2	76660001	76780000	White blood cell number	health traits	immune capacity	6308	1
